# Unveiling acquired resistance to anti-EGFR therapies in colorectal cancer: a long and winding road

**DOI:** 10.3389/fphar.2024.1398419

**Published:** 2024-04-22

**Authors:** Alejandro Ríos-Hoyo, Xavier Monzonís, Joana Vidal, Jenniffer Linares, Clara Montagut

**Affiliations:** ^1^ Yale Cancer Center, Yale School of Medicine, Yale University, New Haven, CT, United States; ^2^ Department of Medical Oncology, Hospital del Mar Research Institute, Centro de Investigación Biomédica en Red de Cáncer (CIBERONC), Barcelona, Spain

**Keywords:** colorectal cancer, acquired resistance, anti-EGFR, liquid biopsy, CtDNA, clonal dynamics, tumor heterogeneity, anti-EGFR rechallenge

## Abstract

Emergence of acquired resistance limits the efficacy of the anti-EGFR therapies cetuximab and panitumumab in metastatic colorectal cancer. In the last decade, preclinical and clinical cohort studies have uncovered genomic alterations that confer a selective advantage to tumor cells under EGFR blockade, mainly downstream re-activation of RAS-MEK signaling and mutations in the extracellular domain of EGFR (EGFR-ECD). Liquid biopsies (genotyping of ctDNA) have been established as an excellent tool to easily monitor the dynamics of genomic alterations resistance in the blood of patients and to select patients for rechallenge with anti-EGFR therapies. Accordingly, several clinical trials have shown clinical benefit of rechallenge with anti-EGFR therapy in genomically-selected patients using ctDNA. However, alternative mechanisms underpinning resistance beyond genomics -mainly related to the tumor microenvironment-have been unveiled, specifically relevant in patients receiving chemotherapy-based multi-drug treatment in first line. This review explores the complexity of the multifaceted mechanisms that mediate secondary resistance to anti-EGFR therapies and potential therapeutic strategies to circumvent acquired resistance.

## 1 Introduction

Colorectal cancer (CRC) represents the third most commonly diagnosed cancer worldwide, and the second leading cause of cancer related deaths (Sung et al., 2021; Siegel et al., 2023). Although lifestyle modifications (e.g., smoking cessation, lowering alcohol intake, increasing dietary fiber, physical activity) can prevent a substantial amount of cases, its estimated incidence is predicted to increase, particularly in countries with a High Development Index (Morgan et al., 2022). Survival rate in metastatic CRC (mCRC) remains poor, with a median overall survival (mOS) of 36 months, and a 5-year OS not exceeding 20% (Cervantes et al., 2022). In the metastatic or unresectable setting, systemic therapy is the treatment of choice, using chemotherapy, targeted therapies, or immunotherapy (Biller and Schrag, 2021; Cervantes et al., 2022). To adequately guide treatment selection in mCRC, biomarker identification is crucial. This involves testing for genomic alterations including *KRAS/NRAS/BRAF mutations*, and microsatellite instability (MSI)/mismatch repair genes (MMR). In this sense, patients with wild-type (WT) *KRAS/NRAS/BRAFV600E* benefit from chemotherapy doublets (FOLFOX [5-Fluouracil, folinic acid and oxaliplatin], CAPOX [capecitabine and oxaliplatin] or FOLFIRI [5-Fluouracil, folinic acid and irinotecan]), combined with EGFR-inhibitors such as cetuximab or panitumumab (Benson et al., 2022; Cervantes et al., 2022).

Cetuximab is an anti-EGFR targeted monoclonal antibody (moAb) that consists of a chimeric immunoglobulin G1 (IgG1), which, upon binding to the EGFR receptor, induces the internalization and degradation of the receptor, thus disrupting the downstream pathway. Since cetuximab is an IgG1 moAb, it can elicit immune functions such as antibody-dependent cell-mediated cytotoxicity (ADCC) as an anti-tumoral effect (Mendelsohn et al., 2015). However, panitumumab, a humanized IgG2 moAb with similar anti-proliferative effects to cetuximab, is not able to initiate an ADCC effect (Yarom and Jonker, 2011). Two decades ago, initial trials of anti-EGFR therapy in mCRC showed the efficacy of cetuximab and panitumumab monotherapy as second and subsequent lines of treatment in patients with WT *KRAS tumors*. These trials reported an objective response rate (ORR) of 12.8% and 17%, a median progression-free survival (mPFS) of 3.7 months and 12.3 weeks, and a median overall survival (mOS) of 9.5 months and 8.1 months, for cetuximab and panitumumab, respectively. Subsequently, the frontline pivotal randomized trials that tested anti-EGFR therapies in combination with chemotherapy doublets (Jonker et al., 2007; Amado et al., 2008; Karapetis et al., 2008), showed a benefit in ORR (46.9%–58%), mPFS (9.9–12 months), and mOS (19.8–24.9 months) (Bokemeyer et al., 2009; 2015; Van Cutsem et al., 2009). In the following years, retrospective analysis of *RAS* (*KRAS, NRAS*) and *BRAF* mutations in tumor tissue samples from patients included in these pivotal trials showed a greater benefit in patients with tumors not harboring *RAS*/*BRAF* mutations.

## 2 Acquired resistance to anti-EGFR moAb in mCRC: heterogeneity and clonal selection

The benefit driven by the addition of anti-EGFR therapy to chemotherapy is undoubtful, however, resistance eventually develops which leads to disease progression. Colorectal tumors are heterogeneous, composed of multiple cellular clones carrying different genetic or epigenetic abnormalities within the same tumor. Understanding this heterogeneity and why tumors are heterogenous is crucial to understand how cancer initiates and evolves, how cancer can be attacked and at the same time how cancer can become resistant to therapy. This heterogeneity may be explained by a branching evolutionary process driven by genetic variation (mainly fostered by genomic instability) and natural selection of the fittest variant driven by microenvironment conditions or external pressures such as therapy (Amirouchene-Angelozzi et al., 2017; Niida et al., 2021). The Darwinian principles of evolution and survival are the basis of tumor heterogeneity and clonal evolution, since the acquisition of different genetic and/or epigenetic alterations endows the tumor with greater survival capabilities, and the capacity to escape drug inhibition (Kreso et al., 2013; Amirouchene-Angelozzi et al., 2017).

Tumor heterogeneity can be studied by sequencing of different regions within a tumor and reconstructing the evolutionary dynamics or the history of a specific cancer, represented in what is called a phylogenetic tree (Siravegna et al., 2018). However, upon metastatic spread and after several lines of drug pressure, heterogeneity becomes greater, and heterogeneity is underrepresented by a single tumor re-biopsy (Gerlinger et al., 2012; Amirouchene-Angelozzi et al., 2017; Dang et al., 2020). A different approach to study tumoral molecular heterogeneity is the use of (serial) liquid biopsies, which are able to detect the genomic landscape shed into the bloodstream by the different subclones (spatial heterogeneity), in a minimally-invasive blood extraction which can be repeated as many times as necessary to track the evolving sub-clonal genetic abnormalities (temporal heterogeneity) (Van Emburgh et al., 2016; Amirouchene-Angelozzi et al., 2017; Dasari et al., 2020; Vidal et al., 2022).

## 3 Translational models to study acquired resistance to EGFR inhibitors

With the goal of finding the best treatment strategies to circumvent or prevent the emergence of acquired resistance to anti-EGFR therapy in the clinical setting, several preclinical studies have been conducted in the last decades to characterize the molecular drivers of acquired resistance to anti-EGFR therapy in CRC. *In vitro* and *in vivo* studies generally include the generation of resistant cells to anti-EGFR therapy by a long-time exposure of cetuximab or panitumumab sensitive cells, followed by molecular characterization of the resistant cells compared to paired initially sensitive cells, and ideally functional studies to confirm causality of the preclinical findings, as well as confirmation of the preclinical findings in tumor samples from patients treated with anti-EGFR therapy. While all studies share these general principles for the generation of drug-resistant cells, each study had its own specificities such as different cancer cell lines (GEO, SW48, DiFi, Lim-1215, CaCo2, NCIH508, OXCO and HCA-46, etc.), or the use of different treatment strategies to generate resistant cells (mainly continuous or increasing exposure of the cells to cetuximab or panitumumab), which may have led to identification of different mechanisms of acquired resistance. These translational models have unveiled a myriad of molecular mechanisms of acquired resistance to anti-EGFR therapy, including c-MET activation, mutations in the extracellular domain of EGFR, mutations in the *RAS* genes (*KRAS* or *NRAS*)*, KRAS* amplification, *PIK3CA* mutation, *ERBB2* amplification and overexpression of EGFR ligands (Ciardiello et al., 2004; Yonesaka et al., 2011; Misale et al., 2012; 2014b; Montagut et al., 2012; Troiani et al., 2013; Hobor et al., 2014; Arena et al., 2015). Table 1 presents different preclinical models of CRC used to study induced resistance to EGFR antibodies.

**TABLE 1:** Preclinical models to induce resistance to anti EGFR antibodies, and the resistance mechanism identified.

Model used	Model characteristics	Intervention	Outcome	Resistance mechanism	Reference
*In vitro*	CaCo2 and Lim 1215 cell lines	Administration of cetuximab at IC_50_ values	Development of cetuximab resistant cell lines	Not identified	De Pauw et al. (2019)
*In vitro* and *in vivo*	Cell line A431 inoculated to immunodeficient mice	Administration of cetuximab	Identification of specimens with no tumor regression, were deemed as resistant	Not identified	Viloria-Petit et al. (2001)
*In vivo*	GEO colon cancer cells inoculated to immunodeficient mice	Administration of cetuximab	Creation of an *in vivo* cetuximab resistant GEO tumor xenograft	MET activation, associated to an overexpression of TGF-α	Ciardiello et al. (2004), Troiani et al. (2013)
*In vitro*	SW48 colon cancer cell line	Continuous exposure to increasing concentrations of cetuximab	Establishing a cetuximab-resistant SW48 cancer cell line		
*In vitro*	DiFi	Administration of cetuximab at a constant dose, or by an increasing exposure, from 3 months to 1 year	Cetuximab-resistant variants	Decrease in *EGFR* gene copy number, and amplification of *KRAS*	Misale et al. (2012)
*In vitro*	LIM 1215			Mutations in *KRAS,* G13D and G12R	Misale et al. (2012)
*In vitro*	Di-Fi cells	Continuous administration of cetuximab for 5 months	DiFi-derived cetuximab-resistant clones	Missense mutation in *EGFR* S492R	Montagut et al. (2012)
*In vitro*	CaCo2 cells	Exposure to increasing concentrations of cetuximab	Caco2 cetuximab resistant cells	Overexpression of long noncoding RNA CRART16	Zhang et al. (2020)
*In vitro*	Di-Fi cells	Continuous exposure to cetuximab for 1 year	Di-Fi cetuximab resistant cells	*KRAS* amplification	Misale et al. (2014a)
	LIM 1215 –1 cells	Continuous exposure to cetuximab for at least 3 months	LIM 1215 cetuximab resistant cells	*KRAS* mutations G12R, K117N and *NRAS* mutation G12C	
	LIM 1215 –2 cells			*KRAS* mutation G13D	
	LIM 1215 –3 cells			*KRAS* mutation A146T	
	LIM 1215 –4 cells			*KRAS* mutations G12D and G13D	
	NCIH508 cells	Continuous exposure to cetuximab for 3–9 months	NCIH508 cetuximab resistant cells	*KRAS* amplification	
	OXCO–2–1 cells		OXCO-2 cetuximab resistant cells	*KRAS* mutation G12D and *BRAF* mutation V600E	
	OXCO–2–2 cells			*NRAS* mutations G12C, G12D and G13D	
	HCA-46 –1 cells		HCA-46 cetuximab resistant cells	*KRAS* amplification	
	HCA-46 –2 cells			*KRAS* mutation G13D	
	Di-Fi cells	Continuous exposure to panitumumab for 3–9 months	Di-Fi panitumumab resistant cells	*KRAS* mutation G12D	
	HCA-46 cells		HCA-46 panitumumab resistant cells	*KRAS* mutation G12C	
	LIM 1215 cells		LIM 1215 panitumumab resistant cells	*KRAS* mutation G13D, and *NRAS* G12C	
	OXCO-2 cells		OXCO-2 panitumumab resistant cells	*KRAS* mutation G12D, and N*RAS* mutation Q61R	
	NCIH508 cells	NA	NCIH508 panitumumab resistant cells	*KRAS* amplification and *NRAS* mutation G12C	
*In vitro*	DiFi cells	Continuous exposure to cetuximab for 1 year	DiFi cetuximab resistant cells	Not reported	Arena et al. (2015)
	OXCO-2 cells	Continuous exposure to cetuximab for 3–9 months	OXCO-2 cetuximab resistant cells	*EGFR* mutation S463L	
	NCIH508 cells		NCIH508 cetuximab resistant cells	*PIK3CA* exon9^a^	
	LIM1215 cells		LIM1215 cetuximab resistant cells	KRAS exon 2, and 3, *NRAS* exon 2, EGFR mutation I491M and G465R	
	HCA-46 cells		HCA-46 cetuximab resistant cells	KRAS exon 2	
	CCK81 cells	Exposure to increasing cetuximab concentrations	CCK81 cetuximab resistant cells	KRAS exon 2, EGFR mutation S464L	
*In vitro*	HCC827 and GEO CRC cells	Exposure to increasing cetuximab concentrations	HCC827 and GEO CRC resistant cells	*ERBB2* amplification	Yonesaka et al. (2011)
*In vitro*	Di-Fi, OXCO-2, and LIM1215 cells	Exposure to increasing cetuximab concentrations	Di-Fi, OXCO-2, and LIM1215 resistant cells	Mutations in *KRAS* G12R, G12D pK117N; *NRAS* G12C; *BRAF* V600E; *KRAS* and *EGFR* amplification. Secretion of TGF-α and amphiregulin	Hobor et al. (2014)

^a^ Not clearly defined as a resistance mechanism.

## 4 Genetic mechanisms of acquired resistance to anti-EGFR therapies

**FIGURE 1:**
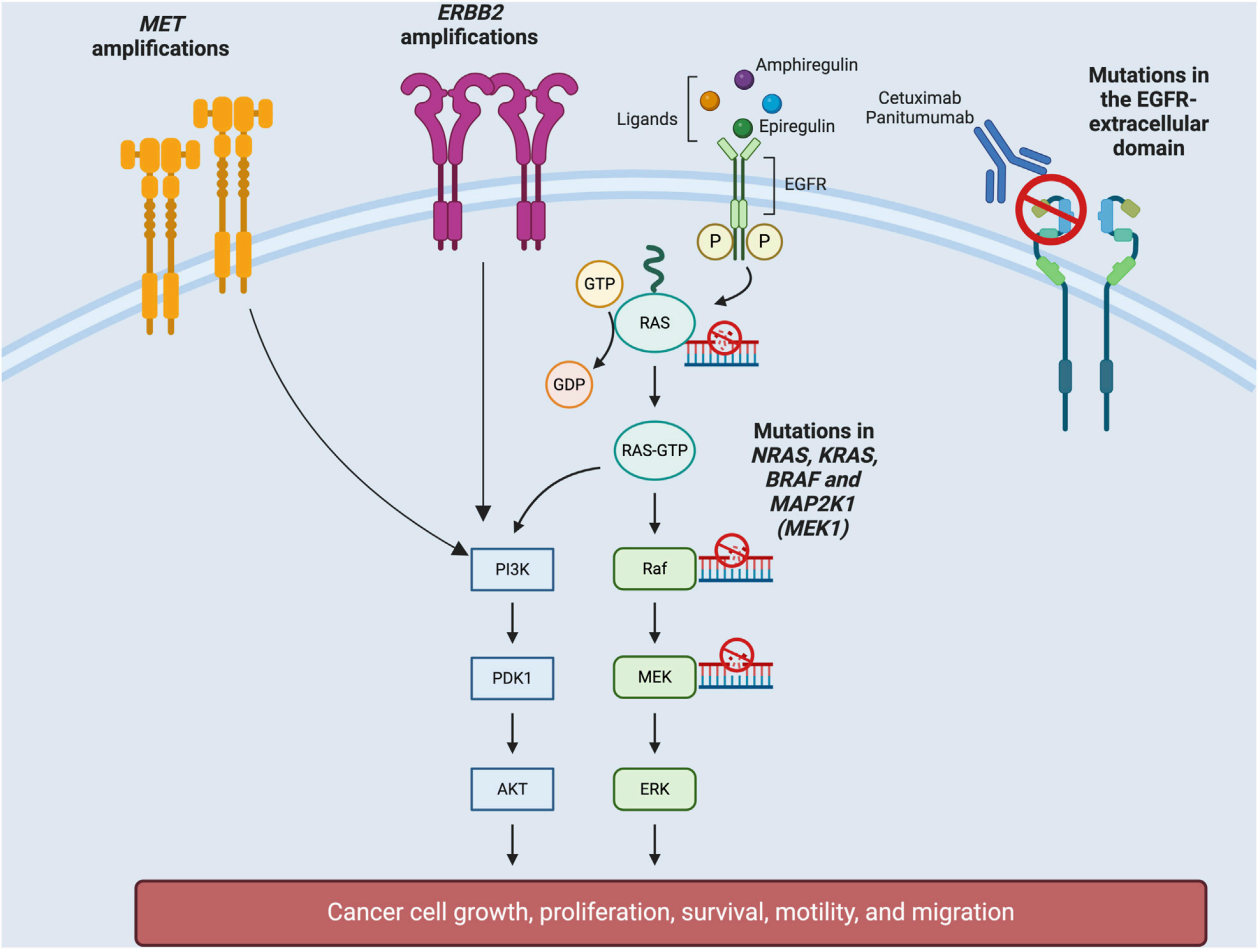
Molecular mechanisms of acquired resistance to anti-EGFR therapies.

Overall, colorectal cancer cells evade EGFR blockade through two main strategies: (a) reactivation of the MAPK-ERK signaling pathway either by mutations in the pathway genes, alterations in alternative tyrosine-kinase receptors, or ligands overexpression (b) lack of binding of cetuximab/panitumumab to the receptor by mutations in the binding epitope located in the extracellular domain of EGFR (Misale et al., 2015; Siravegna et al., 2015). Notably, these mechanisms of resistance can coexist within one same tumor (Figure 1).

### 4.1 RAS alterations

KRAS and NRAS belong to the RAS membrane-bound family proteins, they possess an inherent GTPase activity, and can activate different effector targets, such as the RAF-MAPK, and PI3K-ATK-mTOR pathways (Uprety and Adjei, 2020). Both *KRAS* mutations and amplifications, as well as *NRAS* mutations have been identified as mechanisms of resistance to cetuximab and panitumumab both in liquid biopsy and tumor biopsy specimens (Table 2). The development of these mutations following anti-EGFR targeted treatment, can be a consequence of alterations rising from pre-existent *KRAS* altered clones, or due to new mutations derived from stress conditions induced by targeted therapy to the tumor and tumor microenvironment. Furthermore, it has been reported that several alterations can coexist (Diaz Jr et al., 2012; Misale et al., 2012; Misale et al., 2014a).

**TABLE 2:** Selected mechanisms of acquired resistance to anti-EGFR therapies.

Mechanism of resistance	Alteration	Treatments leading to resistance	Samples where it was identified	References
*KRAS*	Mutations in: G12A, G12C, G12D, G12R, G12V, G13D, G13R, G34A, G34C, G34T, G35A, G35C, G35T, Q61H, Q61K, Q61L, K117N and A1467T	Pmab ± ChT, Cmab ± ChT	Plasma, and tissue re-biopsy	Diaz Jr et al. (2012), Misale et al., 2012 (2014a), Bardelli et al. (2013), Mohan et al. (2014), Arena et al. (2015), Siravegna et al. (2015a), Pietrantonio et al. (2017), Kim et al. (2018), Siena et al. (2018), Bray et al. (2019), Woolston et al. (2019)
*KRAS*	Amplification	Cmab	Plasma, and tissue re-biopsy	Arena et al. (2015), Siravegna et al. (2015a), Woolston et al. (2019)
*NRAS*	G13R, Q61L	Pmab	Plasma, and tissue re-biopsy	Misale et al. (2014a)
*MET*	Amplification	Pmab, Cmab + ChT	Plasma	Bardelli et al. (2013), Mohan et al. (2014), Siravegna et al. (2015a)
*EGFR-ECD*	V441, S464L, G465E, G465R, K467T, S492R	Pmab ± ChT, Cmab ± ChT	Plasma, and tissue re-biopsy	Montagut et al. (2012); Montagut et al. (2018), Arena et al. (2015), Siravegna et al. (2015a), Pietrantonio et al. (2017), Strickler et al. (2018), Parseghian et al. (2019), Woolston et al. (2019), Price et al. (2020)
*ERBB2*	Amplification	Cmab	Plasma	Yonesaka et al. (2011), Mohan et al. (2014), Pietrantonio et al. (2017)
*BRAF*	V600E, D594N	Cmab	Plasma, and tissue re-biopsy	Pietrantonio et al. (2017), Bray et al. (2019), Woolston et al. (2019)
EGF ligands	Low expression of amphiregulin and epiregulin	Cmab and Pmab	Primary tumor biopsy	Jacobs et al. (2009), Seligmann et al. (2016)
Others	Mutations in *AKT1* ^a^, *IDH1* ^a^, *PIK3CA* ^a^, *MAP2K1,* and FGFR1 amplification^a^	Cmab	Tissue re-biopsy	Pietrantonio et al. (2017), Siena et al. (2018), Bray et al. (2019), Parseghian et al. (2023)

^a^ Have not been clearly identified as mechanisms of acquired resistance. Abbreviations used: Cmab: cetuximab, Pmab: panitumumab, ChT: chemotherapy.

### 4.2 *BRAF* mutations

BRAF belongs to the serine/threonine kinases RAF family, its downstream signaling consists of MEK one and two and ERK, leading to further phosphorylation of multiple molecules (Subbiah et al., 2020). Different mutations in the *BRAF* gene have been identified as acquired mechanisms of resistance to anti-EGFR inhibitors, such as V600E and D594N, which lead to a persistent activation on the downstream pathway of RAF-ERK (Pietrantonio et al., 2017; Bray et al., 2019; Woolston et al., 2019).

### 4.3 *ERBB2* amplifications

HER2 belongs to the EGFR tyrosine kinase family, it presents the most potent catalytic kinase activity, and its phosphorylation leads to a downstream activation of the PI3K-AKT-mTOR, and MAPK pathways (Yan et al., 2015; Ríos-Hoyo et al., 2022). *ERBB2* amplifications have been described as resistance mechanisms in plasma samples from patients with acquired resistance to cetuximab, detection in serum of the HER2/HER2 ECD was correlated to resistance to cetuximab at progression. Abnormal activation of HER2 signaling led to persistent ERK 1/2 signaling, induced by treatment with cetuximab (Yonesaka et al., 2011; Mohan et al., 2014).

### 4.4 Mutations in the *EGFR*-extracellular domain (ECD)

Our group identified mutations in the *EGFR-ECD* as a mechanism of resistance to anti-EGFR therapies, these mutations are located in domain III of EGFR, in the binding sites of cetuximab, thus impairing the drug-receptor interaction. The most frequent described *EGFR-ECD* mutations emerging during anti-EGFR therapy are V441, S464, G465, and S492 mutations (Montagut et al., 2018; Strickler et al., 2018). It is worth noting that because the binding epitopes of cetuximab and panitumumab do not fully overlap, some mutations confer resistance to cetuximab but not to panitumumab. This is the case of S492 mutation which does not affect the binding activity of panitumumab, whereas S464L, G465R and 1491M mutations do not allow the binding of neither cetuximab nor panitumumab to the receptor (Montagut et al., 2012; Arena et al., 2015; Price et al., 2020). In this sense, one patient with an S492 mutation after cetuximab treatment responded to treatment with panitumumab monotherapy (Montagut et al., 2012). Importantly, *EGFR-ECD* mutations have not been detected in untreated samples and therefore are thought to drive acquired resistance but not primary resistance. Interestingly, patients who develop mutations in the EGFR-ECD experience greater and more lasting tumor responses con anti-EGFR treatment, compare to patients who develop other mechanisms of resistance, such as RAS mutations (Van Emburgh et al., 2016). This data highlights that the absence of *EGFR-ECD* mutant clones in treatment naïve tumors confers an advantage in terms of the response to anti-EGFR treatment.

### 4.5 *MET* amplifications

The mesenchymal-epithelial transition factor (MET) serves as a transmembrane receptor tyrosine kinase, and it is usually activated by the binding of the hepatocyte growth factor ligands. MET activation further activates other signaling pathways including the RAS-ERK-MAPK, PI3K-AKT-mTOR, Wnt/β-catenin, and STAT pathways (Singh Raghav et al., 2012; Drilon et al., 2017). *MET* amplifications have been detected in plasma samples from patients with acquired resistance to anti-EGFR therapies, methods such as BEAMing and FISH have been used to confirm this finding. It has been suggested that anti-EGFR treatment elicits a selective pressure, and therefore an expansion of preexisting subclones with *MET* amplification. *MET* initiated signaling has been proposed as a mechanism to bypass the EGFR blockade (Bardelli et al., 2013).

## 5 Liquid biopsy to monitor clonal dynamics and track mechanisms of acquired resistance

**FIGURE 2:**
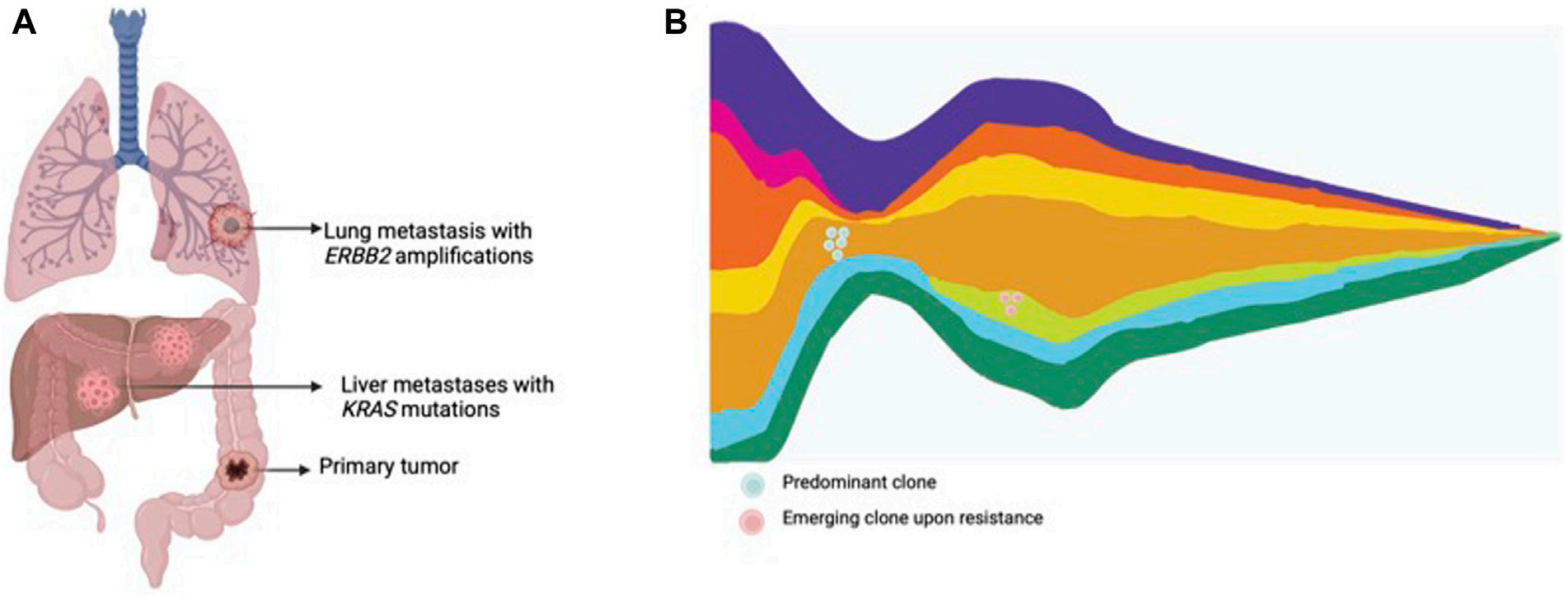
Tumor heterogeneity and clonal dynamics in metastatic CRC. **(A)** Multiple metastatic lesions showing the presence of various subclones within the tumor, each with different genomic alterations. **(B)** Clonal dynamics representing an original dominant clone, responding to treatment, and the rise of other treatment resistant clones.

The term liquid biopsy applied to oncology encompasses the isolation and analysis of tumor derived material in corporal fluids, such as circulating tumor cells, circulating tumor DNA (ctDNA), extracellular vesicles, miRNA, among others. ctDNA is released from tumors into bodily fluids, including blood, cerebrospinal fluid, saliva, pleural fluid, ascites and urine (Wan et al., 2017; Corcoran and Chabner, 2018; Heitzer et al., 2019). Liquid biopsy has been proposed as an exquisite tool to assess intratumor molecular heterogeneity, track clonal dynamics and detect emergent resistant subclones (Wan et al., 2017; Corcoran and Chabner, 2018; Heitzer et al., 2019) (Figure 2). ctDNA is able to comprehensively capture heterogeneity with a high sensitivity for subclones arising under drug pressure. Moreover, the ease-of-use and minimally-invasive procedure allow serial assessment of the genomic landscape to closely track emerging subclones of resistance. In mCRC, several cohort studies and retrospective analysis from clinical trials have shown the utility of liquid biopsy to monitor the genomic landscape and track the emergence of resistant clones in patients treated with anti-EGFR therapies (Diaz Jr et al., 2012; Misale et al., 2012; Siravegna et al., 2015a; Montagut et al., 2018; Vitiello et al., 2019; Dasari et al., 2020; Vidal et al., 2022).

In 2012, the first two studies to show the utility of liquid biopsy in detecting the emergence of *RAS* mutations during treatment with anti-EGFR therapy were concomitantly published (Diaz et al., 2012; Misale et al., 2012). That same year, our group identified the emergence of mutations of acquired resistance in the EGFR extracellular domain during anti-EGFR therapy, which later were also detected in ctDNA. In the following years, other mutations of resistance were detected in ctDNA, including mutations in *BRAF* and *MAP2K1* (Misale et al., 2014b; Siravegna et al., 2015a). Interestingly, using liquid biopsy, our group in collaboration with Bardelli’s group was able to show that not all mutations are the same in regard to treatment response and duration of response. In 27 patients with mCRC, *RAS, EGFR-*ECD and co-occurrence of both mutations were detected in 20, 14 and 7 cases respectively at the time of progression to anti-EGFR therapy. Interestingly, *RAS* mutations were mostly detected in patients who presented stable disease as best response with a shorter duration of response (mPFS of 25.6 weeks), compared to *EGFR*-ECD mutations which were more frequently detected in patients achieving a higher decrease in tumor size (partial response) and a longer duration of response (mPFS of 44.6 weeks). Moreover, *in vitro* studies supported the same concept that *RAS* mutations emerge earlier during anti-EGFR therapy than *EGFR-ECD* mutations (Van Emburgh et al., 2016). It is important to highlight, that multiple mutations of acquired resistance usually co-exist within one same patient after treatment with anti-EGFR therapy, as a consequence of the selection of several clones of resistance (Pietrantonio et al., 2017; Montagut et al., 2018; Strickler et al., 2018) Interestingly, mutation upsurge/emergence of multiple subclones anticipates a remarkable clinical deterioration, especially when *EGFR-ECD* mutations emerge (Toledo et al., 2017; Montagut et al., 2018). Therefore, it may be extremely challenging to pharmacologically target the complex molecular heterogeneity associated with emergence of resistance to cetuximab/panitumumab in mCRC patients.

The use of serial liquid biopsies to track mutations of resistance has showed a decrease in *RAS* and *EGFR-ECD* mutations upon withdrawal of anti-EGFR therapy. Siravegna et al. reported the first study to prove this concept in mCRC patients, in whom *KRAS* mutant alleles, *EGFR-ECD* mutations, and *MET* amplifications detected in ctDNA upon progression to anti-EGFR drugs, diminished and were undetectable several months after finishing anti-EGFR therapy. The intermittent detection of *KRAS* mutant clones in blood of patients treated with anti-EGFR therapies, supports the concept that CRC cells possess an outstanding plasticity (Siravegna et al., 2015a). A similar study showed that the exponential decay of *RAS* and *EGFR-ECD* mutant allele frequency presented a median of 3.4 and 6.9 months, respectively. (Parseghian et al., 2019). Altogether, decay and absence of detection of subclones of resistance few months upon withdrawal of EGFR blockade sets a strong biological rationale for testing clinical strategies of rechallenge with anti-EGFR therapy in mCRC patients.

## 6 Mechanisms of acquired resistance beyond genomics

So far, most studies have focused on genomic alterations as the drivers of acquired resistance to anti-EGFR therapy. However, genomic alterations in EGFR and the MAPKs pathway occur in less than 50% of tumors progressing to anti-EGFR therapy, and recent data show that this percentage is even lower in mCRC patients treated with anti-EGFR therapy plus chemotherapy in the first-line setting. This has led to explore alternative mechanisms of acquired resistance. In an effort to identify novel biomarkers of resistance, transcriptomic profiles from three clinical and two preclinical cohorts treated with cetuximab were used to assign consensus molecular subtypes (CMS) and found excellent responses to cetuximab in CMS2 tumors, independently of primary tumor laterality (Parseghian et al., 2023). Conversely, resistance to anti-EGFR therapy was associated with a transition from CMS2 tumors to CMS4 tumors, characterized by mesenchymal infiltration (Woolston et al., 2019). In this sense, preclinical modeling demonstrated that acquired resistance to either cetuximab or chemotherapy was a result of cross-resistant transcriptomic profiles consistent with epithelial-to-mesenchymal transition. In addition, recent preclinical studies have suggested that anti-EGFR resistance may be driven by cancer associated fibroblasts populating the tumor microenvironment, and their secreted factors (Woolston et al., 2019; Garvey et al., 2020). More recently, data on patients treated in first line and in combination with chemotherapy has revealed novel data and a vastly different profile of mechanisms of resistance to anti-EGFR therapy. Biomarker analysis of the CALBG/SWOG-80405 trial evaluated the development of acquired mechanisms of resistance to anti-EGFR inhibitors using liquid biopsy in patients with metastatic CRC who received a first line treatment with chemotherapy (FOLFOX or FOLFIRI) and cetuximab (n = 61), or chemotherapy (FOLFOX or FOLFIRI) and bevacizumab (n = 69). The authors reported emergence of 6.6% and 10.1% genomic alterations of anti-EGFR resistance in ctDNA at the time of progression to cetuximab and bevacizumab, respectively. Among the reported genomic alterations, mutations in *KRAS, NRAS, BRAF, EGFR-ECD* and amplifications in *ERBB2* and *MET* were reported (Raghav et al., 2023). Parseghian et al. retrospectively analyzed paired ctDNA samples before and after anti-EGFR therapy from three different trials and also demonstrated unique molecular patterns of resistance between first-line and later-line anti-EGFR therapies (Parseghian et al., 2023). Similarly, our group analyzed serial ctDNA samples of patients treated with cetuximab plus chemotherapy in first-line within the PLATFORM-B study, and found that in five out of nine patients with *RAS/BRAF* subclones emerging early (cycle 2) during anti-EGFR plus chemotherapy did not expanded (Vidal et al., 2023). Altogether, these studies suggest that chemotherapy-based multi-drug treatment may favor a specific resistance profile that may include additional mechanisms of resistance (transcriptomic, epigenetic, tumor-microenvironment-derived factors) rather than genomic-driven resistance to the anti-EGFR component of the regimen. Therefore, the use of liquid biopsy to also detect non-genomic alterations of the tumor could provide a comprehensive understanding of tumor evolution during the course of treatment. In addition, understanding the complexity of mechanisms of resistance beyond point mutations of driver genes in cancer cells is crucial to design future successful combination regimens.

## 7 Clinical strategies to overcome resistance

**FIGURE 3:**
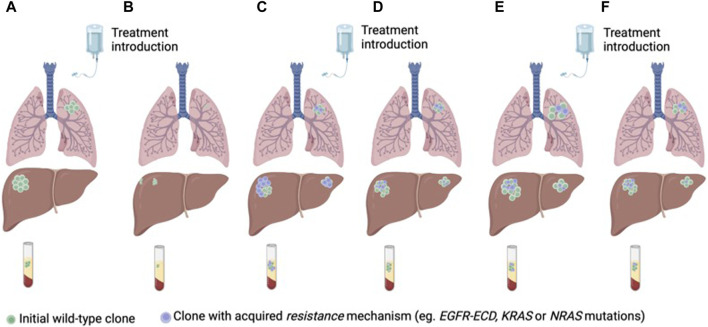
Anti-EGFR treatment of RAS/BRAF wt mCRC according to clonal dynamics assessed in ctDNA **(A)** Treatment naïve CRC with a predominant clone of anti-EGFR sensitive cancer cells **(B)** Tumor response to first-line treatment with anti-EGFR therapy. **(C)** Tumor progression and emergence of sub-clones of resistance to anti-EGFR therapy. **(D)** Decay of the anti-EGFR resistant clones with second-line treatment without anti-EGFR therapy. **(E)** Tumor progression. **(F)** Tumor response to third-line anti-EGFR rechallenge.

Different clinical strategies have been proposed to prevent or circumvent acquired resistance to anti-EGFR therapies. Targeting mutations of resistance is potentially limited by the complex heterogeneity of coexisting subclones of resistance. Another treatment strategy is to take advantage of clonal dynamics and rechallenge with anti-EGFR drugs after a wash-off period and decay of mutations of resistance in ctDNA. Rechallenge refers to the concept of re-treating with anti-EGFR therapy in patients who previously derived a benefit from this drug (Mauri et al., 2019; Martinelli et al., 2020; Mauri et al., 2022) (Figure 3).

Several small phase II clinical trials assessing the efficacy of rechallenge with anti-EGFR therapies have been conducted. In common, all trials include patients that previously responded to anti-EGFR therapy, followed by a subsequent treatment with no anti-EGFR treatment. The first study assessing re-challenge was conducted more than a decade ago and included 39 patients with *KRAS* WT (codons 12 and 13) metastatic CRC re-treated with cetuximab plus irinotecan in third-line. The ORR was 53.8%, and the mPFS 6.6 months (Santini et al., 2012). Following this study, the CRICKET trial was a single-arm phase II study that included 28 patients with metastatic tissue *RAS/BRAF* WT mCRC who previously benefited for at least 6 months of irinotecan-based chemotherapy and cetuximab. The trial aimed to evaluate the activity of these compounds in the third-line setting, and achieved an ORR of 21%, mPFS 3.4 months, and mOS 9.8 months. Importantly, for the first time, the use of ctDNA to select for anti-EGFR rechallenge was retrospectively analyzed. Patients with baseline (before rechallenge) ctDNA *RAS* WT achieved a partial response in 57% of the cases, and had a longer mPFS compared to patients with mutations in *RAS* detected in ctDNA (4 vs. 1.9 months, respectively) (Cremolini et al., 2019). The JACCRO CC-08 trial also evaluated the efficacy of irinotecan plus cetuximab rechallenge in the third-line setting in 34 patients with *KRAS* WT mCRC. One patient achieved a partial response, the disease control rate (DCR) was 55.9%, the mPFS was 2.4 months, and the mOS was 8.2 months. In an attempt to find clinical surrogate markers of clinical benefit, the authors identified that patients with a longer cetuximab free interval (CFI), derived a greater benefit from the rechallenge strategy, as opposed to patients with a short CFI (DCR of 82% vs. 44%, mPFS of 4.6 vs. 2.1 months, and mOS of 14.1 and 6.3 months, respectively for the long and short CFI) (Masuishi et al., 2020). The VELO clinical trial was a randomized trial that evaluated rechallenge with panitumumab plus trifluridine-tipiracil (also known as TAS102) vs. trifluridine-tipiracil alone as control arm. The study included 62 patients with chemo-refractory tissue *RAS* WT mCRC and was positive in favor of the rechallenge strategy (mPFS4 and 2.5 months in panitumumab + trifluridine-tipiracil vs. trifluridine-tipiracil, respectively). Retrospective analysis of baseline ctDNA *RAS/BRAF* WT identified patients obtaining prolonged clinical benefit with panitumumab plus trifluridine-tipiracil compared with trifluridine-tipiracil (PFS rates at 6months 38.5% vs. 13.0% and at 12 months 15.4% vs. 0%). Interestingly, extended ctDNA hyperselection (WT for KRAS, NRAS, BRAFV600E, EGFR ECD, ERBB2, MAP2K1, andPIK3CA) selected patients with a mPFS of 6.4 months, partial response of 13.3% and stable disease of 73.3% (Napolitano et al., 2023). A chemotherapy-free treatment strategy was evaluated in the CAVE trial, a single arm phase II trial that included 77 patients with tissue *RAS* WT mCRC patients rechallenged with cetuximab plus the anti-PD-L1 drug avelumab. The ORR was 7.8%, DCR was 65%, mPFS was 3.6 months, and mOS was 11.6 months. Post-hoc analysis of baseline ctDNA revealed, that patients with *RAS/BRAF* WT ctDNA had a better survival than patients with mutated *RAS/BRAF* (mPFS of 4.1 vs. 3 months, and a mOS of 17.3 vs. 10.4 months, respectively). At progression to rechallenge, ctDNA detected *KRAS/BRAF* and *EGFR-ECD* S292R mutations as mechanisms of resistance (Martinelli et al., 2021)

The CHRONOS clinical trial was the first trial to include genomic selection by ctDNA as an inclusion criterion. Moreover, the trial used a clean design in which panitumumab rechallenge was administered alone to evaluate the effect of anti-EGFR treatment without the effect of concomitant chemotherapy. Panitumumab was administered in 27 patients with *RAS/BRAF* and *EGFR* ECD WT in ctDNA (mutation zero). The study achieved it primary endpoint, with an ORR of 30%, a DCR of 63%, with a median duration of response of 17 weeks, a mPFS of 16 weeks, and a mOS of 55 weeks. Following panitumumab rechallenge, ctDNA identified different resistance mechanisms including mutations or amplifications in *KRAS, NRAS, EGFR, PTEN,* and *MET*, 48% of the patients had at least two co-occurring mechanisms of resistance (Sartore-Bianchi et al., 2021; Sartore-Bianchi et al., 2022). The ongoing CITRIC trial (EudraCT 2020-000443-31) is the first randomized clinical trial aimed to evaluate the efficacy of cetuximab plus irinotecan rechallenge in the third-line setting in comparison to standard treatment at investigator’s choice in patients genomically selected with no detection of mutations of acquired resistance (*RAS, BRAF* and *EGFR-ECD* wild-type) in the blood of patients before rechallenge. Recruitment was recently completed. Table 3 presents different completed and ongoing rechallenge strategies.

**TABLE 3:** Clinical trials using rechallenge strategies with anti-EGFR therapies in patients with colorectal cancer and ctDNA evaluation.

Study	Study design	No. of patients included	Treatment, line and regimen	ctDNA evaluation	Results	Results according to ctDNA
Santini et al. Santini et al. (2012)	Phase II, single arm	39	≥Third-line cetuximab plus irinotecan	No	ORR: 53.8%, mPFS: 6.6 m	NA
CRICKET trial Cremolini et al. (2019)	Phase II single arm	28	Third-line cetuximab and irinotecan	Retrospective analysis of baseline ctDNA	ORR: 21%, mPFS: 3.4 m	mPFS ctDNA *RAS* wt 4 m vs. *RAS* mut 1.9 m
JACCRO CC-08 Sunakawa et al. (2020)	phase II Single arm	34	Third-line Cmab and irinotecan	Retrospective analysis of baseline ctDNA	ORR 2.9% mPFS 2.4 m	Post progression survival after rechallenge was shorter in pts with *RAS* mut
VELO Napolitano et al. (2023)	Phase II randomized	62	TAS102 vs. TAS102 plus Pmab	Retrospective analysis of baseline and end of treatment ctDNA	ORR: 9.7% Pmab + TAS102% vs. 0% TAS102, mPFS:4 m Pmab + TAS102 vs. 2.5 m TAS102	Pmab and TAS102 6 m PFS: ctDNA RAS/BRAF wt. 38% vs. RAS/BRAF mut 13%; 12 m PFS 15.4%vs. 0% respectively
CAVE Martinelli et al. (2021)	phase II Single arm	77	Third line Cmabplus avelumab	Retrospective analysis of baseline ctDNA	ORR:7.8%, mPFS: 3.6 m	mPFS ctDNA *RAS/BRAF/EGFR-ECD* wt 4.1 m vs. RAS/BRAF/EGFR-ECD mut 3 m
CHRONOS Sartore-Bianchi et al. (2022)	phase II Single arm	27	≥ Third-line Pmab	*RAS, BRAF V600E,* and *EGFR-ECD* wt in ctDNA as inclusion criteria	ORR: 30%, mPFS: 16 wks	ctDNA *RAS/BRAF/EGFR-ECD* wt ORR: 30%, mPFS: 16 wks
CITRIC Santos Vivas et al. (2022)	Phase II randomized	58	Third-line Cmab and irinotecan vs. physician’s choice	*RAS, BRAF V600E,* and *EGFR-ECD* wt in ctDNA as inclusion criteria	Recruitment finished	--

Abbreviations: pts: patients, m: months, mCRC: metastatic colorectal cancer, Cmab: cetuximab, Pmab: panitumumab, ChT: chemotherapy, mDoR: median duration of response, wks: weeks, m: months, mut: mutated, wt: wild-type NA: not available.

In a different approach, the Sym004-005 clinical trial evaluated the use of Sym004, a mixture of two synergistic antibodies, futuximab and modotuximab, directed against nonoverlapping epitopes in EGFR, leading to internalization and degradation of the receptor (Sánchez-Martín et al., 2016). A phase II clinical trial evaluated the use of two regimens of Sym004 (higher dose: arm A, lower dose: arm B), compared to chemotherapy (arm C). The study included 254 patients with *KRAS* exon 2 WT mCRC who were refractory to standard chemotherapy and had acquired resistance to anti-EGFR therapies. The mOS was 7.9, 10.3 and 9.6 months for arms A, B and C, respectively. A preplanned retrospective analysis of patients with no detection of mutations in *RAS, BRAF* and *EGFR* ECD in ctDNA, showed a dramatic statistically significant improvement in mOS for treatment with low-dose Sym004 (12.8 vs. 7.3 for the control arm). Again, this study shows the necessity of ctDNA genomic analysis to select patients that benefit from anti-EGFR therapy (Montagut et al., 2018).

## 8 Conclusion

Therapeutic anti-EGFR moAbs (cetuximab and panitumumab) remain the mainstay of targeted therapy in RAS/BRAF wild-type metastatic colorectal cancer. However, resistance eventually develops leading to cancer progression. In the last decade, preclinical and translational models have identified two main strategies for colorectal cancer cells to evade EGFR inhibition: reactivation of the MAPK pathway and mutations in the extracellular domain of EGFR (EGFR ECD). These genomic alterations arise as a consequence of heterogeneity and clonal selection under drug pressure. Interestingly, liquid biopsy (i.e., genotyping of ctDNA) is a minimally invasive method to track genomic alterations of resistance in the blood of patients treated with cetuximab/panitumumab. Treatment of resistance to anti-EGFR therapies remains a challenge, since genomic alterations of resistance are multiple and coexist within one same tumor. Because mutations of acquired resistance decline over time following anti-EGFR withdrawal, an alternative strategy that is showing promising results in several phase II clinical trials is to rechallenge with anti-EGFR therapy in patients selected by no detection of mutations of acquired resistance in liquid biopsy. More recently, alternative mechanisms of resistance beyond genomics, mainly related to the tumor microenvironment, have been identified, specifically in patients treated with chemotherapy-based multi-drug treatment in first line of treatment (vs. anti-EGFR single treatment in heavily pretreated patients). In the era of personalized medicine, it is of the utmost importance to better understand the complexity of the mechanisms of acquired resistance to anti-EGFR therapy to be able to design appropriate clinical trials and ultimately improve treatment and care of mCRC patients.
